# The effect of different methods to identify, and scenarios used to address energy intake misestimation on dietary patterns derived by cluster analysis

**DOI:** 10.1186/s12937-021-00696-3

**Published:** 2021-05-08

**Authors:** Geraldine Lo Siou, Alianu K. Akawung, Nathan M. Solbak, Kathryn L. McDonald, Ala Al Rajabi, Heather K. Whelan, Sharon I. Kirkpatrick

**Affiliations:** 1 Cancer Research & Analytics, Alberta Health Services, Richmond Road Diagnostic & Treatment Centre, 1820 Richmond Rd SW, Calgary, Alberta T2T 5C7 Canada; 2 Health Sciences Department, College of Natural and Health Sciences, Zayed University, Abu Dhabi, UAE; 3 Department of Health and Physical Education, Faculty of Health, Community and Education, Mount Royal University, Calgary, AB Canada; 4 School of Public Health and Health Systems, University of Waterloo, Waterloo, ON Canada

**Keywords:** Alberta’s tomorrow project, Cluster analysis, Dietary patterns, Energy intake, Misreporting, Predicted total energy expenditure method, Revised-Goldberg method

## Abstract

**Background:**

All self-reported dietary intake data are characterized by measurement error, and validation studies indicate that the estimation of energy intake (EI) is particularly affected.

**Methods:**

Using self-reported food frequency and physical activity data from Alberta’s Tomorrow Project participants (*n* = 9847 men 16,241 women), we compared the revised-Goldberg and the predicted total energy expenditure methods in their ability to identify misreporters of EI. We also compared dietary patterns derived by *k*-means clustering under different scenarios where misreporters are included in the cluster analysis (Inclusion); excluded prior to completing the cluster analysis (ExBefore); excluded after completing the cluster analysis (ExAfter); and finally, excluded before the cluster analysis but added to the ExBefore cluster solution using the nearest neighbor method (InclusionNN).

**Results:**

The predicted total energy expenditure method identified a significantly higher proportion of participants as EI misreporters compared to the revised-Goldberg method (50% vs. 47%, *p* < 0.0001). *k*-means cluster analysis identified 3 dietary patterns: Healthy, Meats/Pizza and Sweets/Dairy. Among both men and women, participants assigned to dietary patterns changed substantially between ExBefore and ExAfter and also between the Inclusion and InclusionNN scenarios (Hubert and Arabie’s adjusted Rand Index, Kappa and Cramer’s V statistics < 0.8).

**Conclusions:**

Different scenarios used to account for EI misreporters influenced cluster analysis and hence the composition of the dietary patterns. Continued efforts are needed to explore and validate methods and their ability to identify and mitigate the impact of EI misestimation in nutritional epidemiology.

**Supplementary Information:**

The online version contains supplementary material available at 10.1186/s12937-021-00696-3.

## Background

Over the past two decades, nutrition research has transitioned from a focus on single nutrients or foods, to a more holistic approach, describing overall eating patterns [1–3]. It is now appreciated that foods and beverages are consumed in combination and there are synergistic and potentially antagonist interactions among components of an individuals’ overall diet [4–6]. This challenges our ability to attribute health effects to individual dietary components [7]. Dietary pattern analysis acknowledges the complexity of dietary intake, with the potential to provide improved estimates of disease risk [7].

Several methods for examining dietary patterns exist, including cluster analysis [8, 9]. Although other clustering methods exist, such as the Ward’s or flexible beta methods, our previous work using a split-half cross-validation approach showed that *k*-means clustering produced cluster solutions with the highest reproducibility [10]. Other research supports the use of *k*-means clustering methods by showing the stability of the dietary patterns produced [9].

Epidemiological studies involving dietary pattern analysis utilize self-reported dietary intake data obtained from tools such as food frequency questionnaires (FFQs), food records and 24-h dietary recalls [11], due to their feasibility and cost-effectiveness. However, dietary data collected from such tools are error-prone [12, 13]. This error can mask true estimates of diet-disease associations, leading to untrustworthy conclusions about diet-disease relationships [14]. It is important for epidemiological studies using self-reported dietary data to acknowledge error, consider optimal strategies to mitigate it, and to carefully report these details to allow appropriate interpretation of findings [15].

Energy intake is particularly affected by error, likely because errors in reporting of each food and beverage consumed, compounds when assessing total energy intake [12]. In cohort studies, “true” energy intake (EI) cannot be readily assessed because the doubly labeled water (DLW) method, which is purported to be an unbiased marker of EI, is prohibitively expensive [16]. A reliance on self-reported data continues within epidemiologic research. There is often interest in assessing plausibility of estimated EI based on reported food and beverage consumption [17].

A commonly used crude method is to exclude participants who report fewer than 500 and greater than 3, 500 cal per day [17], for example, prior to conducting analysis. This method is not individualized and may not identify all implausible reports of EI, as well as potentially excluding some individuals with plausible EI estimates [17]. Alternatively, the ratio of reported energy intake (rEI) to predicted energy requirement may be calculated and this value included in statistical models to adjust for misreporting [17]. However, this approach assumes that foods and beverages are misreported proportionately, which may not be true [17].

More sophisticated statistical methods, such as the revised-Goldberg [18] and the predicted total energy expenditure (pTEE) methods [19, 20], have been developed to assess the plausibility of estimated EI in relation to energy expenditure (EE). Compared to other methods, the revised-Goldberg and pTEE methods make use of more parameters, including basal metabolic rate (BMR) and physical activity level (PAL), and are individualized [17]. These statistical methods can identify those who are affected by misestimation of total energy intake with reasonable accuracy. The sensitivity and specificity of the revised-Goldberg method in comparison to DLW in a random sample of men and women, aged 40–69 years, in the Washington, D.C. metropolitan area was 92 and 88%, respectively using the NCI Diet History Questionnaire [21]. Jessri et al. [22] suggest that the pTEE method is currently the most detailed statistical procedure for identifying EI misreporters [19, 20].

Few studies have compared the revised-Goldberg and pTEE methods in their ability to account for the plausibility of EI [23, 24] and their implications for analyses conducted within nutritional epidemiology. In particular, it remains unclear if the revised-Goldberg and pTEE methods lead to comparable identification of misestimation of EI and how the choice of statistical method to account for misestimation influences dietary pattern outcomes. The objectives of this study were (i) to compare the revised-Goldberg and pTEE methods in terms of their ability to identify EI misreporters (EI-MR), and (ii) to compare dietary patterns derived by *k*-means clustering under different scenarios of accounting for EI-MR.

## Methods

### Study population

We drew upon data from Alberta’s Tomorrow Project (ATP), a longitudinal cohort of ~ 55,000 Albertans established in 2000, providing a research platform to study the etiology of cancer and chronic diseases. Albertans aged 35–69 years, with no personal history of cancer except non-melanoma skin cancer, were recruited into ATP. Study design, participant recruitment, enrollment and data collection methods are described in detail elsewhere [25–27].

The current analyses were restricted to participants (*n* = 26,814) who completed a baseline Health and Lifestyle Questionnaire (HLQ) [27], Canadian Diet History Questionnaire-I (CDHQ-I) [28] and a validated Past Year Total Physical Activity Questionnaire (PYTPAQ) [29] at enrollment between 2000 and 2008. Participants who were recruited as “second in household” (*n* = 342), reported at enrollment a personal history of cancer (except non-melanoma skin using Alberta Cancer Registry data; *n* = 69), were categorized as underweight based on self-reported body weight and standing height (*n* = 181), had missing information on body weight, standing height or age (*n* = 71), and pregnant women at enrollment (*n* = 63) were excluded from this analysis. The final sample size was *n* = 26,088 participants (median age (IQR), 50.0 (14.0) years, 37.8% men).

### Sociodemographic and anthropometric measures

Information on participant’s age, sex, educational attainment, annual household income, tobacco use, body weight and standing height were collected at the time of enrollment using the HLQ, which was developed by the ATP cohort researchers using a combination of existing questions from other large-scale studies [27]. BMI was calculated from self-reported standing height and body weight.

### Dietary intake assessment

Dietary intake data were collected using the CDHQ-I, a 257-item past-year FFQ of foods, beverages and dietary supplements, based on the US National Cancer Institute’s Diet History Questionnaire and modified for use in Canada [28, 30]. Past year dietary intake data collected using CDHQ-I were analyzed using Diet*Calc software (version 1.4.2; National Cancer Institute), and the CDHQ-I nutrient database was used to estimate average daily intakes of energy, 66 nutrients and 284 single foods and dietary supplements. Based on similarities in macronutrient composition and culinary use, the 284 single food items were combined into 55 food groups [10]. The daily percentage of EI contributed by each of the 55 food groups was used as input variables in the *k*-means cluster analysis.

### Physical activity assessment

Physical activity was assessed using an accelerometer-validated PYTPAQ [29], showing acceptable reliability (*r* = 0.64) and validity (ICC = 0.41), to collect information on the frequency, duration and intensity of recreational, household, transport and occupational physical activities during the past year. Physical activity level (PAL) was calculated as the ratio of energy expenditure (EE) to basal metabolic rate (BMR). Specifically, EE was calculated using the following equation [31]:

$$ {\displaystyle \begin{array}{c} EE=\left[ BMR-\right( total\ average\ time\ spent\ performing\ recreational, household,\\ {} transport\ and\ occupational\ activities\ in\ hours\  per\  day\ast body\ weight\ in\  kg\Big)\\ {}\begin{array}{c}+\Big( total\ average\ metabolic\ output\ from\ recreational, household, transport\ and\\ {} occupational\ activities\ in\  MET\  hours\  per\  day\ast body\ weight\ in\  kg\left)\right]\ast 1.1\end{array}\end{array}} $$

BMR was calculated from participant’s body weight, standing height, age and sex using the Mifflin equation [32]:

$$ {\displaystyle \begin{array}{c} BMR\ \left(\frac{kcal}{day}\right)=9.99\ast body\ weight\ in\  kg+6.25\ast standing\ height\ in\  cm\\ {}-4.92\ast age+166\ast sex\left( males,1; females,0\right)-161\end{array}} $$

PAL was categorized into four groups: sedentary (1.0 ≤ PAL< 1.4), low active (1.4 ≤ PAL< 1.6), active (1.6 ≤ PAL< 1.9), and very active (PAL≥1.9).

### EI misreporters identification

#### Revised-Goldberg method

Details of the method were described originally by Goldberg et al. [33], then revised by Black [18]. In brief, assuming that body weight is stable and EI equals EE [16], the revised-Goldberg method assesses the plausibility of rEI by comparing the ratio of rEI to BMR (rEI:BMR) to the ratio of EE to BMR (EE:BMR, also known as PAL) [18]. The method estimates 95% confidence limits of the agreement between rEI:BMR and PAL, with the following equation:

$$ cut\  off\ {points}_{Revised- Goldberg}={PAL}_{value}\ast \exp \left(\pm 2\ast \frac{\left(\sqrt{\frac{CV_{rEI}^2}{d}+{CV}_{BMR}^2+{CV}_{PAL}^2}\right)}{100}\right) $$

where PAL_value_ is the assigned PAL_value_ specified by the Institute of Medicine [34] for each group of PAL (PAL_value_ = 1.25, 1.50, 1.75 and 2.20 if sedentary, low active, active, and very active, respectively), CV_rEI_ is the intra-individual variation in rEI, d is the number of dietary assessments completed, CV_BMR_ is the intra-individual variation in repeated BMR measurements or the precision of estimated compared with measured BMR, and CV_PAL_ is inter-individual variation in PAL.

Based on suggestions from Black [18], the following values were used in the above equation: CV_BMR_ = 8.5%, CV_PAL_ = 15%. Since the present study used FFQ to assess dietary intake, the values for CV_rEI_ suggested by Tooze et al. [21] were used: CV_rEI_ = 19.8% for women and CV_rEI_ = 18.6% for men. Since only one FFQ was completed in the present study and the input variable for the cluster analysis was the average daily rEI, d was chosen to be equal to 1 [21, 35]. To account for the skewness in the distribution of energy intake, 95% confidence intervals for rEI:BMR were estimated on a logarithmic scale. Individuals with the natural log transformation of (rEI:BMR) below, above, and within the cut-off points were identified as EI-UR, EI-OR, and EI plausible reporters (EI-PR), respectively. The revised-Goldberg cut-offs used in this analysis were: lower = 0.75 and upper = 2.08 for sedentary, lower = 0.90 and upper = 2.49 for low active, lower = 1.05 and upper = 2.91 for active, and lower = 1.32 and upper = 3.65 for very active.

#### Predicted Total energy expenditure (pTEE) method

The pTEE method was originally developed by McCrory et al. [19]. Briefly, rEI is compared with pTEE using a TEE prediction equation based on DLW. Huang et al. [20] modified the method by comparing rEI with estimated energy requirements (EER), which are derived from TEE prediction equations from the Institute of Medicine based on a DLW [36]. The following equations are used for individuals aged 19 years or older:

Men with BMI 18.5–24.9 kg/m^2^:

$$ {\displaystyle \begin{array}{c} EER\ \left(\frac{kcal}{day}\right)=662-\left(9.53\ast age\  in\ years\right)+{PAL}_{coefficient}\\ {}\ast \left(15.91\ast body\ weight\ in\  kg+539.6\ast standing\ height\ in\ m\right)\end{array}} $$

where PAL_coefficient_ = 1 if sedentary, PAL_coefficient_ = 1.11 if low active, PAL_coefficient_ = 1.25 if active, and PAL_coefficient_ = 1.48 if very active.

Women with BMI 18.5–24.9 kg/m^2^:

$$ {\displaystyle \begin{array}{c} EER\ \left(\frac{kcal}{day}\right)=354-\left(6.91\ast age\  in\ years\right)+{PAL}_{coefficient}\\ {}\ast \left(9.36\ast body\ weight\ in\  kg+726\ast standing\ height\ in\ m\right)\end{array}} $$

where PAL_coefficient_ = 1 if sedentary, PAL_coefficient_ = 1.12 if low active, PAL_coefficient_ = 1.27 if active, and PAL_coefficient_ = 1.45 if very active.

Men with BMI ≥25 kg/m^2^:

$$ {\displaystyle \begin{array}{c} EER\ \left(\frac{kcal}{day}\right)=1086-\left(10.1\ast age\  in\ years\right)+{PAL}_{coefficient}\\ {}\ast \left(13.7\ast body\ weight\ in\  kg+416\ast standing\ height\ in\ m\right)\end{array}} $$

where PAL_coefficient_ = 1 if sedentary, PAL_coefficient_ = 1.12 if low active, PAL_coefficient_ = 1.29 if active, and PAL_coefficient_ = 1.59 if very active.

Women with BMI ≥25 kg/m^2^:

$$ {\displaystyle \begin{array}{c} EER\ \left(\frac{kcal}{day}\right)=448-\left(7.95\ast age\  in\ years\right)+{PAL}_{coefficient}\\ {}\ast \left(11.4\ast body\ weight\ in\  kg+619\ast standing\ height\ in\ m\right)\end{array}} $$

where PAL_coefficient_ = 1 if sedentary, PAL_coefficient_ = 1.16 if low active, PAL_coefficient_ = 1.27 if active, and PAL_coefficient_ = 1.44 if very active.

Similar to the revised-Goldberg method, the pTEE method estimates confidence intervals for the ratio (rEI:EER), and 1.0 or 2.0 SD cut-off points are calculated. However, to compare with the revised-Goldberg method, 2.0 SD cut-off points were used in the present study, with the following equation:

$$ {cutoff\ points}_{pTEE}=\pm 2\ast \left(\sqrt{\frac{CV_{rEI}^2}{d}+{CV}_{EER}^2+{CV}_{mTEE}^2}\right) $$

where CV_rEI_ is the intra-individual variation in rEI, d is the number of dietary assessments completed, CV_EER_ is the error in the equations for EER, and CV_mTEE_ is the day-to-day biological variation and the measurement error for TEE based on the DLW method [37].

Based on suggestions from Huang et al. [20], the following values were used in the above equation: CV_EER_ = 11.0%, CV_mTEE_ = 8.2%. Since the present study used an FFQ to assess dietary intake, the values for CV_rEI_ were 19.8% for women and 18.6% for men [21]. Since only one FFQ was completed and the input variable for the cluster analysis was the average daily rEI, d was set to 1 [21, 35]. To account for the skewness in the distribution of energy intake, the 95% confidence interval for rEI:EER was estimated on a logarithmic scale and cut-off points were exponentiated [22, 38]. The ratio (rEI:EER) was expressed as a percentage and individuals with (%rEI:EER) below, above, and within the cut-off points were identified as EI-UR, EI-OR, and EI-PR, respectively. The pTEE cut-offs used in this analysis were: lower = 0.79 and upper = 1.26 for normal weight, and lower = 0.63 and upper = 1.59 for overweight or obesity.

### Statistical analysis

**Fig. 1:**
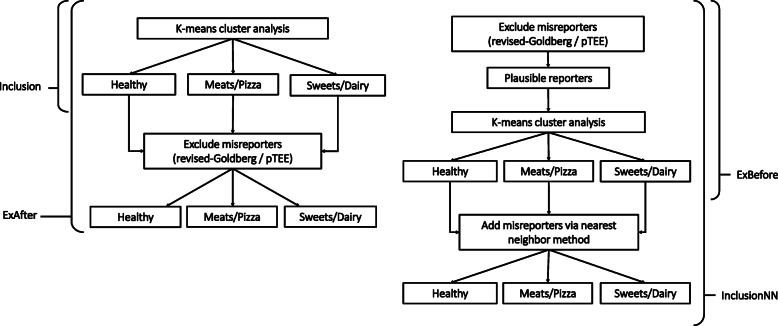
Flow chart illustrating different scenarios to account for potential misreporting of energy intake ExBefore, Energy intake misreporters are excluded prior to completing the cluster analysis, ExAfter, Energy intake misreporters are excluded after completing the cluster analysis, Inclusion, Energy intake misreporters are included in the cluster analysis, InclusionNN, Energy intake misreporters are excluded before the cluster analysis but added to the ExBefore cluster solution using the nearest neighbor method.

*k*-means cluster analysis [39] was performed separately for men and women, with the number of clusters varying from 2 to 7 to balance feasibility and robustness [15]. To explore the potential effect of EI-MR on cluster analysis, four scenarios were examined: inclusion of EI-MR in cluster analysis (Inclusion) [15]; exclusion prior to completing the cluster analysis (ExBefore) [15]; exclusion after completing the cluster analysis (ExAfter) [15]; and exclusion before the cluster analysis but adding EI-MR back to the ExBefore cluster solution using the nearest neighbor method (InclusionNN) [15, 40] (Fig. 1). The nearest neighbor method (*k* = 1) is a pattern classification method that measures the Euclidean distance between a test example (i.e., participant) and the data set and assigns the test example to the cluster of the nearest neighbor [40].

To reduce the impact of local optima, *k*-means cluster analysis was repeated 10 times with different starting seeds for each cluster solution. The cluster solution with the minimum total within-cluster sum of squares distances was selected [9]. Then for each selected cluster solution, the natural log-transformed ratio of between-versus within-cluster variances were calculated and compared using boxplots. To ensure heterogeneity among clusters, the cluster solution with the highest natural log-transformed ratio of between-versus within-cluster variances and with many food groups assigned to each cluster was chosen as optimal [10]. With increasing cluster solutions (5 or more), the number of food groups assigned to each cluster decreased and the solution could no longer be considered to reflect a dietary pattern. As a result, only cluster solutions ranging from 2 to 4 were considered.

Before cluster analysis, each input variable was standardized using the range method [41]. Food groups were assigned to the cluster to which they contributed the highest rEI. Labels were established based on cluster assignment of mutually exclusive food groups to form dietary patterns.

The proportions of EI-MR in both the Inclusion and InclusionNN scenarios were compared across dietary patterns using the chi-square test. Kappa, Cramer’s V and Hubert and Arabie’s adjusted Rand Index [42] were used to measure the agreement in the assignment of participants to the dietary patterns between ExBefore and ExAfter and between Inclusion and InclusionNN cluster solutions. Values ≥0.8 were considered in good agreement, indicating that dietary patterns assigned to participants did not substantially change between the two compared scenarios [43].

Descriptive statistics were presented as medians and interquartile ranges (IQR) for continuous variables, and as frequencies and percentages for categorical variables. Kappa statistic was calculated to assess the agreement in the identification of EI-MR and EI-PR between the revised-Goldberg and pTEE methods. The Pearson chi-square test was used to examine differences in the proportions of EI-MR between the revised-Goldberg and pTEE methods.

All analyses were conducted using SAS Enterprise Guide, version 7.13 (SAS Institute Inc., Cary, NC, USA), and the criterion for statistical significance was set as alpha ≤0.05 (two-tailed).

## Results

### Participant characteristics at enrollment by EI reporting status

The participant characteristics at enrollment are presented in Table 1. The majority of participants were women, completed post-secondary education, employed full-time, and married or living with a partner.

**Table 1:** Participant characteristics at enrollment, by EI reporting status based on the revised-Goldberg and pTEE methods

Characteristics	Revised-Goldberg		pTEE		
	EI-MR	EI-PR	EI-MR	EI-PR	Total
	(*n* = 12,333)	(*n* = 13,755)	(*n* = 13,153)	(*n* = 12,935)	(*n* = 26,088)
	47.3%	52.7%	50.4%	49.6%	
Sex					
Men, n (%)	4719 (38.3)	5128 (37.3)	5096 (38.7)	4751 (36.7)	9847 (37.8)
Women, n (%)	7614 (61.7)	8627 (62.7)	8057 (61.3)	8184 (63.3)	16,241 (62.3)
Age in years, median (IQR)	50.0 (14.0)	50.0 (15.0)	50.0 (15.0)	50.0 (14.0)	50.0 (14.0)
Marital status, n (%)					
Married/With partner	9467 (76.8)	11,034 (80.2)	10,117 (76.9)	10,384 (80.3)	20,501 (78.6)
Single	736 (6.0)	770 (5.6)	794 (6.0)	712 (5.5)	1506 (5.8)
Divorced/Separated/Widowed	2129 (17.3)	1949 (14.2)	2240 (17.0)	1838 (14.2)	4078 (15.6)
Educational attainment, n (%)					
Post-secondary completed	6126 (49.7)	7299 (53.1)	6611 (50.3)	6814 (52.7)	13,425 (51.5)
Some post-secondary	2659 (21.6)	2693 (19.6)	2811 (21.4)	2541 (19.7)	5352 (20.5)
High school completed	2353 (19.1)	2475 (18.0)	2470 (18.8)	2358 (18.2)	4828 (18.5)
High school not completed	1194 (9.7)	1287 (9.4)	1260 (9.6)	1221 (9.4)	2481 (9.5)
Employment status, n (%)					
Employed full-time	7371 (59.8)	7243 (52.7)	7734 (58.8)	6880 (53.2)	14,614 (56.0)
Employed part-time	1902 (15.4)	2475 (18.0)	2032 (15.5)	2345 (18.1)	4377 (16.8)
Not employed	1538 (12.5)	2030 (14.8)	1646 (12.5)	1922 (14.9)	3568 (13.7)
Retired	1519 (12.3)	2001 (14.6)	1738 (13.2)	1782 (13.8)	3520 (13.5)
Annual household income, n (%)					
< $50,000	3814 (31.6)	4228 (31.5)	4028 (31.3)	4014 (31.8)	8042 (31.6)
$50,000–$99,999	5087 (42.2)	5597 (41.7)	5420 (42.1)	5264 (41.8)	10,684 (41.9)
> $100,000	3165 (26.2)	3584 (26.7)	3418 (26.6)	3331 (26.4)	6749 (26.5)
Smoking Status, n (%)					
Current smoker	2043 (16.6)	2492 (18.1)	2170 (16.5)	2365 (18.3)	4535 (17.4)
Former smoker	4805 (39.0)	5027 (36.6)	5122 (39.0)	4710 (36.4)	9832 (37.7)
Never smoked	5471 (44.4)	6233 (45.3)	5848 (44.5)	5856 (45.3)	11,704 (44.9)
Body mass index in kg/m^2^, n (%)					
18.0–24.9	3333 (27.0)	5447 (39.6)	3754 (28.5)	5026 (38.9)	8780 (33.7)
25.0–29.9	4959 (40.2)	5348 (38.9)	5242 (39.9)	5065 (39.2)	10,307 (39.5)
≥ 30.0	4041 (32.8)	2960 (21.5)	4157 (31.6)	2844 (22.0)	7001 (26.8)
Physical Activity Level, median (IQR)	2.0 (0.5)	1.8 (0.5)	2.0 (0.5)	1.9 (0.6)	1.9 (0.5)
EI in kcal/day, median (IQR)	1327 (573)	2104 (884)	1322 (534)	2152 (847)	1702 (918)
%EI from carbohydrate, median (IQR)	51.0 (11.1)	49.7 (10.6)	51.0 (11.1)	49.6 (10.5)	50.3 (10.9)
%EI from protein, median (IQR)	16.0 (3.8)	15.9 (3.6)	16.0 (3.8)	15.9 (3.6)	15.9 (3.7)
%EI from fat, median (IQR)	31.8 (9.0)	33.5 (8.9)	31.8 (9.0)	33.6 (8.9)	32.7 (9.1)
%EI from alcohol, median (IQR)	1.4 (3.7)	1.2 (3.7)	1.4 (3.7)	1.1 (3.7)	1.3 (3.7)
Dairy in servings/day, median (IQR)	1.0 (1.1)	1.7 (1.7)	1.0 (1.0)	1.8 (1.7)	1.4 (1.4)
Fruit in servings/day, median (IQR)	2.0 (1.9)	2.8 (2.6)	1.9 (1.8)	2.9 (2.7)	2.4 (2.3)
Vegetable in servings/day, median (IQR)	3.1 (2.2)	4.6 (2.9)	3.1 (2.1)	4.7 (2.9)	3.8 (2.8)
Whole grain in servings/day, median (IQR)	0.8 (0.8)	1.3 (1.1)	0.8 (0.8)	1.3 (1.1)	1.0 (1.0)

*EI-MR* Energy Intake Misreporters, *EI-PR* Energy Intake Plausible Reporters, *IQR* Interquartile Range, *pTEE* Predicted Total Energy Expenditure

The revised-Goldberg method identified 46 and 53% of participants as EI-UR and EI-PR, respectively, while the pTEE method identified 50% of participants as EI-UR and EI-PR, respectively. Both the revised-Goldberg and the pTEE methods identified only 1% of the study sample as EI-OR (data not shown). Agreement in the classification of participants as EI-UR, EI-OR, and EI-PR between the two methods was high (Kappa = 0.88; 95% Confidence Interval: 0.87–0.88). EI-OR comprised a very small proportion of the study sample. Therefore, the EI-UR and EI-OR groups were collapsed into EI-MR to provide a sufficient sample size for subsequent analyses (EI-MR revised-Goldberg: 47%, EI-MR pTEE: 50%, *p* < 0.0001).

### The choice of optimal number of clusters

**Fig. 2:**
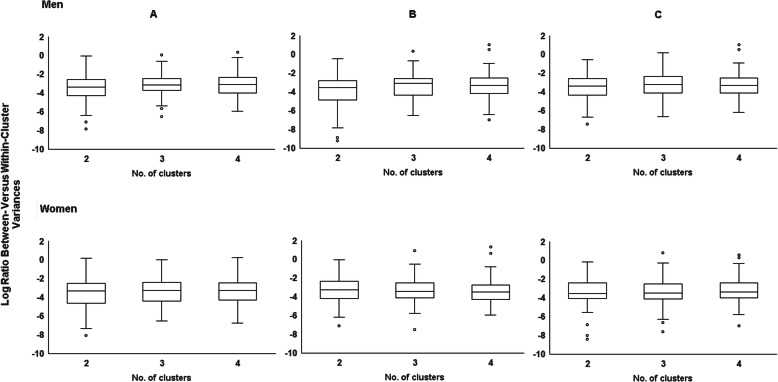
Comparison of natural log-ratio between- versus within-cluster variances in the 2-, 3-, and 4-cluster solutions when **a** Including Misreporters; **b** Excluding Misreporters (Revised-Goldberg Method); **c** Excluding Misreporters (pTEE Method) and stratified by sex

In men, the median log-ratio value of the between-versus within-cluster variances was highest for the 3-cluster solution in all men and men identified as PR using both methods for assessing misestimation of EI (Fig. 2). The 3-cluster solution was therefore chosen as the optimal number of clusters and labeled as “Healthy”, “Meats/Pizza”, and “Sweets/Dairy” to reflect commonalities in food groups included in each cluster (Additional file 1).

For all women and women identified as EI-PR, the median log-ratio values of the between-versus within-cluster variances varied little across the different cluster solutions. The choice of optimal number of clusters in women was therefore based on identifying a cluster solution with many food groups assigned to each cluster so that dietary patterns contained a substantial number of the 55 food groups. The 3-cluster solution was chosen as the optimal cluster solution in women because it resulted in patterns with many food groups in each cluster (Fig. 2). The clusters were labeled as “Healthy”, “Meats/Pizza”, and “Sweets/Dairy” to reflect commonalities in food groups included in each cluster (Additional file 2). Results for cluster solutions greater than four are reported in Additional files 1 and 2.

### Proportion of EI misreporters across dietary patterns

Table 2 presents the proportions of EI-MR across dietary patterns in the Inclusion and InclusionNN scenarios, respectively, based on the revised-Goldberg and pTEE methods. For the Inclusion scenario, higher proportions of EI-MR were found in the Healthy pattern compared to the Meats/Pizza and Sweets/Dairy patterns in both men and women, using the revised-Goldberg and pTEE methods.

**Table 2:** Proportions of EI misreporters (EI-MR) across dietary patterns in the Inclusion and InclusionNN scenarios for men and women, based on the revised-Goldberg and pTEE methods

	Inclusion Scenario		InclusionNN Scenario	
Clusters	Revised-Goldberg	pTEE	Revised-Goldberg	pTEE
	EI-MR	EI-MR	EI-MR	EI-MR
***Men***	**(*n*** **= 4719) %**	**(*n*** **= 5096) %**	**(*n*** **= 4719) %**	**(*n*** **= 5096) %**
Healthy	55.2	60.0	48.7	53.8
Meats/Pizza	44.8	48.0	43.4	46.2
Sweets/Dairy	45.6	49.4	53.4	56.9
***Women***	**(*n*** **= 7614) %**	**(*n*** **= 8057) %**	**(*n*** **= 7614) %**	**(*n*** **= 8057) %**
Healthy	51.4	54.3	48.2	50.4
Meats/Pizza	45.5	47.4	45.6	47.1
Sweets/Dairy	44.3	47.9	47.4	53.1

*EI-MR* Energy Intake Misreporters, *Inclusion* Energy intake misreporters are included in the cluster analysis, *InclusionNN*, Energy intake misreporters are excluded before the cluster analysis but added to the ExBefore cluster solution using the nearest neighbor method, *pTEE* Predicted Total Energy Expenditure

For the InclusionNN scenario, higher proportions of EI-MR were found in the Sweets/Dairy pattern in men, using both methods. In women, higher proportions of EI-MR were found in the Healthy pattern using the revised-Goldberg method while higher proportions of EI-MR were found in the Sweets/Dairy pattern using the pTEE method.

### Agreement among cluster assignments

Table 3 presents the Hubert and Arabie’s adjusted Rand index, Kappa and Cramer’s V statistics for ExBefore vs. ExAfter and Inclusion vs. InclusionNN, respectively, based on the revised-Goldberg and pTEE methods. In both methods, for men and women, the values were each < 0.8. This indicates that the assignment of participants to dietary patterns changed substantially between the ExBefore and ExAfter scenarios and also between the Inclusion and InclusionNN scenarios.

**Table 3:** Hubert and Arabie’s Rand index, Kappa and Cramer’s V statistics for men and women identified as EI-PR (ExBefore vs. ExAfter) and all men and women (Inclusion vs. InclusionNN) based on the revised-Goldberg and pTEE methods

**Agreement between ExBefore and ExAfter**					
**Revised-Goldberg**			**pTEE**		
Rand Index^a^	Kappa^b^	Cramer’s V^c^	Rand Index^a^	Kappa^b^	Cramer’s V^c^
*EI-PR Men (n = 5128)*			*EI-PR Men (n = 4751)*		
0.34	0.53	0.57	0.33	0.52	0.57
*EI-PR Women (n = 8627)*			*EI-PR Women (n = 8184)*		
0.53	0.71	0.71	0.44	0.63	0.64
**Agreement between Inclusion and InclusionNN**					
**Revised-Goldberg**			**pTEE**		
Rand Index^a^	Kappa^b^	Cramer’s V^c^	Rand Index^a^	Kappa^b^	Cramer’s V^c^
*All Men (n = 9847)*			*All Men (n = 9847)*		
0.34	0.53	0.57	0.33	0.52	0.57
*All Women (n = 16,241)*			*All Women (n = 16,241)*		
0.53	0.71	0.71	0.44	0.63	0.64

*EI-PR* Energy Intake Plausible Reporters, *ExBefore* Energy intake misreporters are excluded prior to completing the cluster analysis, *ExAfter* Energy intake misreporters are excluded after completing the cluster analysis, *Inclusion* Energy intake misreporters are included in the cluster analysis, *InclusionNN* Energy intake misreporters are excluded before the cluster analysis but added to the ExBefore cluster solution using the nearest neighbor method, *pTEE* Predicted Total Energy Expenditure

^a^ Hubert and Arabie’s adjusted Rand index is a modified version of the Rand index that determines the similarity between 2 cluster assignments by counting the number of pairwise agreements and disagreements between cluster assignments. Hubert and Arabie’s adjusted Rand index can take negative values, and its upper bound is 1. The closer the Hubert and Arabie’s adjusted Rand index’s positive values are to 1, the better the agreement between cluster assignments

^b^ Kappa statistic is a measure of interrater agreement and is used in this study as a measure of agreement between cluster assignments. Kappa statistic generally ranges between 0 and 1, although its lower bound can be negative if the observed probability of agreement is less than the expected one. Complete agreement is encountered when the Kappa statistic equals 1; therefore, it should be maximized

^c^ Cramer’s V statistic measures the strength of association between cluster assignments and varies between 0 and 1, except in the case of 2 clusters where values range from −1 to 1. Cramer’s V statistic should have values far away from 0, as values closer to − 1 or 1 indicate stronger association between cluster assignments

### Dietary patterns in relation to methods of accounting for misreporting of energy intake

The food groups contributing the greatest proportions to daily EI (> 1%), across dietary patterns and scenarios of accounting for EI misreporting using the revised-Goldberg and pTEE methods are presented in Tables 4 and 5.

**Table 4:** Percentage contribution of food groups to energy intake across dietary patterns and different methods to account for misreporting of energy intake based on the revised-Goldberg and pTEE methods among men

**Healthy Pattern**							
	**MEN**						
		**revised-Goldberg**			**pTEE**		
	**Inclusion**^**a**^	**ExBefore**^**b**^	**ExAfter**^**c**^	**InclusionNN**^**d**^	**ExBefore**^**b**^	**ExAfter**^**c**^	**InclusionNNd**
	(*n* = 2690)	(*n* = 1780)	(*n* = 1205)	(*n* = 3468)	(*n* = 1551)	(*n* = 1076)	(*n* = 3359)
**Food Groups**	Means^e^ (SD)	Means^e^ (SD)	Means^e^ (SD)	Means^e^ (SD)	Means^e^ (SD)	Means^e^ (SD)	Means^e^ (SD)
	%	%	%	%	%	%	%
Fruit	9.9 (5.4)	7.8 (5.0)	9.3 (5.2)	8.1 (5.4)	7.9 (4.9)	9.2 (5.2)	8.3 (5.4)
Low-fat dairy	n/a^f^	6.0 (6.7)	n/a^f^	5.9 (6.8)	5.6 (6.4)	n/a^f^	5.5 (6.3)
Breakfast cereal	4.6 (4.1)	4.2 (3.4)	4.2 (3.5)	4.4 (3.8)	4.1 (3.4)	4.1 (3.5)	4.4 (3.8)
Fruit juice	4.5 (5.4)	4.5 (5.6)	4.6 (5.7)	4.4 (5.4)	4.6 (5.8)	4.8 (5.9)	4.4 (5.4)
Rice	3.6 (6.0)	3.3 (5.7)	4.0 (6.4)	3.1 (5.5)	3.3 (5.5)	3.9 (6.2)	3.1 (5.4)
Nuts	3.1 (5.0)	3.2 (4.9)	3.7 (5.5)	2.7 (4.6)	3.3 (5.1)	3.8 (5.6)	2.8 (4.7)
Poultry no skin	3.0 (3.5)	2.9 (3.4)	3.2 (3.7)	2.8 (3.3)	3.0 (3.5)	3.3 (3.8)	2.8 (3.2)
Regular fat dairy	2.7 (3.2)	2.1 (2.6)	2.6 (2.9)	2.2 (2.9)	2.3 (2.5)	2.6 (2.7)	2.4 (3.0)
Cooked vegetables	1.9 (1.7)	1.7 (1.6)	2..0 (1.8)	1.6 (1.5)	1.7 (1.6)	2.0 (1.8)	1.6 (1.5)
Soup	1.8 (2.1)	1.7 (1.9)	1.8 (2.1)	1.7 (2.0)	1.6 (1.9)	1.7 (2.0)	1.6 (1.9)
Fish	1.6 (1.6)	1.4 (1.5)	1.6 (1.6)	1.4 (1.4)	1.5 (1.5)	1.6 (1.6)	1.5 (1.5)
Wine	1.5 (3.3)	1.4 (3.4)	1.6 (3.5)	1.4 (3.3)	1.4 (3.1)	1.6 (3.6)	1.4 (3.1)
Legumes	1.2 (1.6)	1.1 (1.2)	1.2 (1.3)	1.1 (1.5)	1.1 (1.2)	1.3 (1.4)	1.1 (2.5)
Meal Replacement	1.2 (4.3)	1.1 (4.5)	1.5 (5.3)	1.0 (4.0)	1.2 (4.6)	1.6 (5.5)	1.0 (3.9)
**Meats / Pizza Pattern**							
	**MEN**						
		**revised-Goldberg**			**pTEE**		
	**Inclusion**^**a**^	**ExBefore**^**b**^	**ExAfter**^**c**^	**InclusionNN**^**d**^	**ExBefore**^**b**^	**ExAfter**^**c**^	**InclusionNN**^**d**^
	(*n* = 3924)	(*n* = 2127)	(*n* = 2165)	(*n* = 3760)	(*n* = 2036)	(*n* = 2039)	(*n* = 3786)
**Food Groups**	Means^e^ (SD)	Means^e^ (SD)	Means^e^ (SD)	Means^e^ (SD)	Means^e^ (SD)	Means^e^ (SD)	Means^e^ (SD)
	%	%	%	%	%	%	%
Meat	11.6 (5.4)	10.6 (5.4)	11.6 (5.4)	10.3 (5.4)	10.3 (5.4)	11.6 (5.4)	10.0 (5.4)
Pasta/pizza	6.8 (4.7)	6.8 (4.8)	6.9 (4.9)	6.7 (4.6)	6.7 (4.7)	6.9 (4.9)	6.6 (4.5)
Beer	5.6 (11.0)	5.2 (10.8)	5.8 (11.1)	5.0 (11.0)	5.2 (10.7)	5.8 (11.1)	5.0 (10.9)
Regular soda	4.3 (6.4)	5.0 (7.2)	4.5 (6.7)	4.7 (6.9)	5.1 (7.2)	4.5 (6.8)	4.8 (6.9)
Chips	3.6 (3.6)	3.9 (3.7)	3.6 (3.5)	3.8 (3.8)	3.9 (3.7)	3.6 (3.5)	3.8 (3.7)
Other breads	3.5 (3.7)	n/a^f^	3.5 (3.8)	n/a^f^	n/a^f^	3.5 (3.8)	n/a^f^
Processed meat	3.5 (2.6)	3.4 (2.6)	3.5 (2.6)	3.3 (2.6)	3.4 (2.6)	3.5 (2.6)	3.3 (2.6)
Regular fat cheese	2.4 (2.8)	2.6 (2.8)	2.5 (2.8)	2.4 (2.7)	2.5 (2.8)	2.5 (2.8)	2.4 (2.7)
French fries	2.3 (2.2)	2.2 (2.0)	2.3 (2.2)	2.1 (2.1)	2.1 (2.0)	2.3 (2.1)	2.1 (2.0)
Confectionery	n/a^f^	2.2 (3.0)	n/a^f^	2.1 (2.9)	2.2 (3.0)	n/a^f^	2.0 (3.0)
Eggs	2.2 (2.1)	n/a^f^	2.0 (1.8)	n/a^f^	n/a^f^	2.0 (1.8)	n/a^f^
Liquor	1.9 (5.0)	1.9 (5.3)	1.9 (5.1)	1.9 (5.1)	1.9 (5.6)	1.9 (5.1)	1.9 (5.3)
Regular fat salad dressing	1.5 (2.0)	1.5 (1.9)	1.5 (1.9)	1.5 (1.9)	1.5 (1.9)	1.5 (1.9)	1.5 (2.0)
Mexican	1.3 (1.4)	1.2 (1.6)	n/a^f^	1.3 (1.6)	1.2 (1.6)	n/a^f^	1.2 (1.5)
Butter	1.3 (1.8)	1.2 (1.8)	1.3 (1.9)	1.2 (1.8)	1.3 (1.9)	1.3 (1.9)	1.3 (1.9)
High-fat dairy	1.2 (3.4)	n/a^f^	1.3 (3.5)	n/a^f^	n/a^f^	1.3 (3.5)	n/a^f^
**Sweets / Dairy Pattern**							
	**MEN**						
		**revised-Goldberg**			**pTEE**		
	**Inclusion**^**a**^	**ExBefore**^**b**^	**ExAfter**^**c**^	**InclusionNN**^**d**^	**ExBefore**^**b**^	**ExAfter**^**c**^	**InclusionNN**^**d**^
	(*n* = 3233)	(*n* = 1221)	(*n* = 1758)	(*n* = 2619)	(*n* = 1164)	(*n* = 1636)	(*n* = 2702)
**Food Groups**	Means^e^ (SD)	Means^e^ (SD)	Means^e^ (SD)	Means^e^ (SD)	Means^e^ (SD)	Means^e^ (SD)	Means^e^ (SD)
	%	%	%	%	%	%	%
Low fat dairy	7.3 (7.5)	n/a^f^	7.2 (7.3)	n/a^f^	n/a^f^	7.2 (7.3)	n/a^f^
Whole meal bread	5.0 (4.9)	4.8 (4.6)	4.9 (4.5)	4.5 (4.8)	4.7 (4.6)	4.9 (4.5)	4.5 (4.8)
Jam	4.8 (4.5)	5.0 (4.7)	4.8 (4.5)	4.5 (4.6)	4.9 (4.6)	4.8 (4.5)	4.5 (4.5)
Cake	4.7 (4.3)	3.9 (4.1)	5.1 (4.6)	3.5 (3.7)	4.0 (4.2)	5.2 (4.7)	3.5 (3.7)
Other bread	n/a	3.5 (4.2)	n/a^f^	3.4 (4.1)	3.5 (4.2)	n/a^f^	3.4 (4.2)
Cooked potatoes	3.1 (2.6)	3.2 (2.3)	2.9 (2.3)	3.2 (2.6)	3.3 (2.4)	2.9 (2.3)	3.3 (2.7)
Dessert	2.2 (2.3)	1.9 (2.0)	2.2 (2.3)	1.8 (1.9)	1.9 (2.0)	2.2 (2.3)	1.8 (2.0)
Confectionery	2.2 (3.2)	n/a^f^	2.3 (3.4)	n/a^f^	n/a^f^	2.3 (3.4)	n/a^f^
Margarine	1.9 (2.1)	2.5 (2.4)	1.9 (2.1)	2.1 (2.3)	2.6 (2.4)	1.9 (2.1)	2.1 (2.3)
Eggs	n/a^f^	2.2 (2.0)	n/a^f^	2.3 (2.3)	2.1 (2.0)	n/a^f^	2.2 (2.3)
Ice cream	1.8 (2.6)	1.6 (2.4)	1.9 (2.6)	1.5 (2.3)	1.6 (2.4)	2.0 (2.6)	1.5 (2.2)
Coffee	1.3 (1.2)	1.8 (0.8))	1.0 (0.9)	2.1 (1.2)	1.7 (0.8)	1.0 (0.8)	2.1 (1.2)
High-fat dairy	n/a^f^	1.6 (3.9)	n/a^f^	1.4 (3.7)	1.6 (4.0)	n/a^f^	1.5 (3.7)

*pTEE* Predicted Total Energy Expenditure

^a^ Inclusion reports on all participants. Misreporters were included in the *k*-means cluster analysis. ^b^ ExBefore reports on plausible reporters; however, exclusion of misreporters was completed before *k*-means cluster analysis. ^c^ ExAfter reports on plausible reporters; however, exclusion of misreporters was completed after *k*-means cluster analysis. ^d^ InclusionNN reports on all participants; however, misreporters identified by either revised-Goldberg or pTEE methods are excluded before cluster analysis but added to the ExBefore cluster solution using the nearest neighbor method. ^e^ Mean percentage contribution by each food group. ^f^ Not applicable, indicating that food groups were not assigned to a given dietary pattern if their mean percentage contribution to total rEI was not the highest in that dietary pattern

**Table 5:** Percentage contribution of food groups to energy intake across dietary patterns and different methods to account for misreporting of energy intake based on the revised-Goldberg and pTEE methods among women

**Healthy Pattern**							
	**WOMEN**						
		**revised-Goldberg**			**pTEE**		
	**Inclusion**^**a**^	**ExBefore**^**b**^	**ExAfter**^**c**^	**InclusionNN**^**d**^	**ExBefore**^**b**^	**ExAfter**^**c**^	**InclusionNN**^**d**^
	(*n* = 4808)	(*n* = 2919)	(*n* = 2339)	(*n* = 5633)	(*n* = 3831)	(*n* = 2197)	(*n* = 4933)
**Food Groups**	Means^e^ (SD)	Means^e^ (SD)	Means^e^ (SD)	Means^e^ (SD)	Means^e^ (SD)	Means^e^ (SD)	Means^e^ (SD)
	%	%	%	%	%	%	%
Fruit	13.3 (6.3)	11.6 (6.0)	12.9 (6.0)	11.6 (6.5)	12.1 (5.9)	12.9 (6.0)	12.0 (6.5)
Regular fat dairy	5.1 (4.6)	4.4 (3.9)	4.9 (4.1)	4.4 (4.3)	4.3 (3.9)	4.8 (4.1)	4.4 (4.3)
Poultry no skin	4.6 (4.6)	4.3 (4.2)	4.6 (4.4)	4.3 (4.4)	4.3 (4.1)	4.6 (4.4)	4.2 (4.4)
Nuts	3.5 (5.5)	4.2 (6.1)	4.4 (6.3)	3.4 (5.3)	4.3 (6.0)	4.5 (6.4)	3.5 (5.3)
Rice	3.0 (3.7)	3.1 (3.8)	3.2 (3.9)	3.0 (3.9)	3.2 (3.9)	3.2 (4.0)	3.1 (4.0)
Whole meal bread	n/a^f^	3.2 (3.2)	n/a^f^	3.2 (3.3)	n/a^f^	n/a^f^	n/a^f^
Cooked vegetables	2.6 (2.3)	2.5 (2.3)	2.6 (2.4)	2.4 (2.2)	2.6 (2.3)	2.6 (2.3)	2.5 (2.3)
Soup	1.9 (2.2)	1.9 (2.1)	1.9 (2.0)	2.0 (2.3)	1.9 (1.9)	1.9 (1.9)	1.9 (2.1)
Fish	1.9 (2.2)	1.9 (2.0)	1.9 (2.1)	1.9 (2.1)	1.9 (2.0)	1.9 (2.1)	1.9 (2.2)
Wine	1.7 (3.4)	1.8 (3.7)	1.7 (3.6)	1.7 (3.6)	1.6 (3.3)	1.6 (3.5)	1.6 (3.1)
Raw vegetables	1.5 (1.1)	1.4 (0.9)	1.4 (0.9)	1.4 (1.1)	1.4 (0.9)	1.4 (0.9)	1.5 (1.1)
Legumes	1.5 (1.6)	1.5 (1.5)	1.5 (1.6)	1.5 (1.6)	1.6 (1.6)	1.6 (1.6)	1.5 (1.6)
Cabbage	1.3 (1.6)	1.2 (1.3)	1.2 (1.4)	1.2 (1.5)	1.2 (1.4)	1.2 (1.4)	1.3 (1.6)
Meal replacement	1.1 (3.9)	1.0 (3.6)	1.2 (3.9)	1.0 (3.7)	1.1 (3.8)	1.2 (4.1)	1.0 (3.8)
**Meats / Pizza Pattern**							
	**WOMEN**						
		**revised-Goldberg**			**pTEE**		
	**Inclusion**^**a**^	**ExBefore**^**b**^	**ExAfter**^**c**^	**InclusionNN**^**d**^	**ExBefore**^**b**^	**ExAfter**^**c**^	**InclusionNN**^**d**^
	(*n* = 6643)	(*n* = 3835)	(*n* = 3621)	(*n* = 7049)	(*n* = 2448)	(*n* = 3492)	(*n* = 7245)
**Food Groups**	Means^e^ (SD)	Means^e^ (SD)	Means^e^ (SD)	Means^e^ (SD)	Means^e^ (SD)	Means^e^ (SD)	Means^e^ (SD)
	%	%	%	%	%	%	%
Meat	9.2 (4.8)	8.6 (4.7)	9.2 (4.7)	8.4 (4.8)	8.5 (4.6)	9.3 (4.8)	8.3 (4.7)
Pasta/pizza	6.5 (4.4)	6.2 (4.2)	6.4 (4.3)	6.2 (4.3)	5.9 (4.1)	6.4 (4.3)	5.9 (4.2)
Chips	3.8 (4.0)	3.8 (4.0)	3.9 (4.1)	3.7 (4.0)	3.6 (3.9)	3.9 (4.2)	3.5 (3.9)
Regular soda	3.5 (6.6)	3.5 (6.7)	3.6 (6.7)	3.4 (6.6)	3.5 (6.6)	3.6 (6.8)	3.3 (6.5)
Other breads	3.4 (3.6)	3.1 (3.2)	3.3 (3.4)	3.1 (3.4)	3.1 (3.3)	3.3 (3.4)	3.1 (3.4)
Jam	n/a^f^	2.8 (2.9)	n/a^f^	2.7 (3.0)	2.8 (2.8)	n/a^f^	2.7 (2.9)
Cake	n/a^f^	3.3 (3.4)	n/a^f^	3.1 (3.2)	n/a^f^	n/a^f^	n/a^f^
Cooked potatoes	2.8 (2.2)	2.7 (2.0)	2.7 (2.0)	2.8 (2.2)	2.6 (1.9)	2.7 (2.0)	2.7 (2.2)
Regular fat cheese	2.7 (3.3)	2.7 (3.2)	2.7 (3.2)	2.6 (3.2)	2.8 (3.2)	2.7 (3.1)	2.7 (3.2)
Processed meat	2.5 (1.9)	2.4 (1.8)	2.5 (1.9)	2.4 (1.9)	2.4 (1.8)	2.5 (1.9)	2.4 (1.9)
Confectionery	2.5 (3.7)	2.7 (4.0)	2.6 (3.9)	2.5 (3.8)	2.6 (3.9)	2.6 (3.9)	2.5 (3.7)
Eggs	2.2 (2.4)	2.1 (2.1)	2.1 (2.1)	2.1 (2.3)	2.1 (2.1)	2.1 (2.0)	2.1 (2.3)
Regular fat salad dressing	2.1 (2.7)	2.0 (2.5)	2.1 (2.6)	2.1 (2.7)	2.1 (2.6)	2.1 (2.6)	2.1 (2.7)
Dessert	1.7 (1.9)	1.8 (2.0)	1.8 (1.9)	1.8 (2.0)	1.8 (2.1)	1.8 (1.9)	1.7 (2.0)
Margarine	1.6 (2.1)	1.7 (2.1)	1.7 (2.1)	1.6 (2.1)	1.7 (2.1)	1.7 (2.1)	1.7 (2.1)
French fries	1.5 (1.7)	1.4 (1.6)	1.4 (1.6)	1.4 (1.7)	1.4 (1.6)	1.4 (1.6)	1.4 (1.7)
Butter	1.5 (2.2)	1.7 (2.4)	1.6 (2.3)	1.5 (2.3)	1.7 (2.4)	1.6 (2.3)	1.5 (2.4)
Mexican	1.3 (1.4)	1.2 (1.4)	1.2 (1.4)	1.3 (1.4)	1.2 (1.3)	1.2 (1.4)	1.2 (1.3)
Beer	1.3 (4.5)	1.3 (4.5)	1.2 (4.2)	1.3 (4.8)	1.3 (4.4)	1.2 (4.2)	1.3 (4.8)
Ice cream	n/a^f^	1.2 (2.0)	n/a^f^	1.2 (1.9)	n/a^f^	n/a^f^	n/a^f^
Coffee	1.2 (1.2)	1.1 (0.9)	n/a^f^	1.3 (1.3)	1.1 (0.9)	n/a^f^	1.3 (1.3)
Liquor	1.1 (3.4)	1.1 (3.6)	1.1 (3.6)	1.0 (3.5)	1.1 (3.5)	1.1 (3.5)	1.1 (3.5)
High-fat dairy	1.1 (3.1)	1.2 (3.4)	1.2 (3.4)	1.1 (3.3)	1.1 (3.2)	1.2 (3.4)	1.1 (3.1)
**Sweets / Dairy Pattern**							
	**WOMEN**						
		**revised-Goldberg**			**pTEE**		
	**Inclusion**^**a**^	**ExBefore**^**b**^	**ExAfter**^**c**^	**InclusionNN**^**d**^	**ExBefore**^**b**^	**ExAfter**^**c**^	**InclusionNN**^**d**^
	(*n* = 4790)	(*n* = 1873)	(*n* = 2667)	(*n* = 3559)	(*n* = 1905)	(*n* = 2495)	(*n* = 4062)
**Food Groups**	Means^e^ (SD)	Means^e^ (SD)	Means^e^ (SD)	Means^e^ (SD)	Means^e^ (SD)	Means^e^ (SD)	Means^e^ (SD)
	%	%	%	%	%	%	%
Low fat dairy	10.3 (8.1)	14.3 (6.5)	10.3 (7.6)	13.3 (7.7)	9.2 (7.7)	10.4 (7.6)	8.6 (7.9)
Breakfast cereal	5.1 (4.2)	5.0 (3.7)	4.6 (3.5)	5.2 (4.3)	6.9 (2.7)	4.5 (3.4)	7.2 (3.8)
Whole meal bread	4.5 (4.3)	n/a^f^	4.5 (4.0)	n/a^f^	3.4 (3.3)	4.4 (3.9)	3.3 (3.5)
Fruit juice	4.2 (5.6)	3.8 (4.7)	4.3 (5.5)	3.7 (4.7)	3.9 (4.8)	4.3 (5.5)	3.7 (4.7)
Cake	3.4 (3.4)	n/a^f^	3.7 (3.7)	n/a^f^	n/a^f^	3.7 (3.7)	n/a^f^
Jam	2.9 (3.0)	n/a^f^	3.0 (2.9)	n/a^f^	n/a^f^	3.0 (2.9)	n/a^f^
Ice cream	1.2 (1.9)	n/a^f^	1.2 (3.0)	n/a^f^	n/a^f^	1.3 (1.9)	n/a^f^

*pTEE* Predicted Total Energy Expenditure

^a^ Inclusion reports on all participants. Misreporters were included in the k-means cluster analysis. ^b^ ExBefore reports on plausible reporters; however, exclusion of misreporters was completed before k-means cluster analysis. ^c^ ExAfter reports on plausible reporters; however, exclusion of misreporters was completed after k-means cluster analysis. ^d^ InclusionNN reports on all participants; however, misreporters identified by either revised-Goldberg or pTEE methods are excluded before cluster analysis but added to the ExBefore cluster solution using the nearest neighbor method. ^e^ Mean percentage contribution by each food group. ^f^ Not applicable, indicating that food groups were not assigned to a given dietary pattern if their mean percentage contribution to total rEI was not the highest in that dietary pattern

Among men, several food groups assigned to each dietary pattern changed across the different scenarios to account for EI misreporting, using both the revised-Goldberg and pTEE methods. For example, ‘Low-Fat Dairy’ was not included in the Healthy pattern within the Inclusion and ExAfter scenarios, but was assigned to the Sweets/Dairy pattern for these scenarios. In the Meats/Pizza pattern, ‘Other Breads’, ‘Confectionery’, ‘Eggs’ and ‘High-Fat Dairy’ were not consistently present across the different scenarios (Table 4). Similarly, ‘Low-Fat Dairy’, ‘Other Breads’, ‘Confectionery’, ‘Eggs’ and ‘High-Fat Dairy’ were not consistently present across the different scenarios in the Sweets/Dairy pattern. The assignment of food groups to each dietary pattern was consistent when comparing equivalent scenarios between the two methods of identifying EI-MRs.

Among women, the food groups assigned to each dietary pattern also changed across scenarios. ‘Whole Meal Bread’ was included in the Healthy pattern only within the ExBefore and InclusionNN scenario based on the revised-Goldberg method (Table 5). ‘Jam’, ‘Cake’, ‘Ice Cream’ and ‘Coffee’ were not consistently present across the different scenarios in the Meats/Pizza pattern. Likewise, ‘Whole Meal Bread’, ‘Jam’, ‘Cake’ and ‘Ice Cream’ were not consistently present across scenarios in the Sweets/Dairy pattern. The assignment of food groups to each dietary pattern was inconsistent between the revised-Goldberg and pTEE methods when comparing equivalent scenarios between the two methods of identifying EI-MRs. For example, ‘Whole meal bread’ was included in the Healthy pattern within the InclusionNN and ExBefore scenarios based on the revised-Goldberg method but excluded from the respective scenarios based on the pTEE method.

## Discussion

Our findings suggest that misestimation of EI was prevalent among adult participants. The pTEE method identified a significantly higher proportion of participants as EI-MR compared to the revised-Goldberg method. Different methods and scenarios to account for this misestimation appeared to impact the composition of dietary patterns as some food groups were included in a dietary pattern within certain scenarios but not in others. The composition of the Meats / Pizza and Sweets / Dairy patterns appeared to be most affected by different scenarios for both men and women. Between scenarios, the percentage contributions of each food group differed, causing food groups to be inconsistently assigned to these mutually exclusive dietary patterns. The choice of method appeared to alter the assignment of food groups to dietary patterns in women but not in men.

Other studies have used similar approaches to identify and account for EI misestimation in dietary intake data. A prospective cohort study of American nurses aged 30–55 years [24] compared the Goldberg and the pTEE methods in accounting for misreporting EI in epidemiological studies. Similar to the current study, this study reported higher proportion of EI-MR using the pTEE method (33.8%) compared to the Goldberg method (31.3%). Although the current study compared the pTEE to the revised-Goldberg and not the original Goldberg method, the American study excluded women with obesity, so errors in BMR estimates using the Schofield equation were minimized. Hence, the main findings are not expected to change even if the revised-Goldberg method were used [24]. A Spanish cohort study compared different methods to account for plausibility of dietary intake data including the revised-Goldberg, and the pTEE methods [23]. Unlike the current study, the Spanish study reported higher proportions of EI-UR using the revised-Goldberg method (9.1 and 14.4% in men and women, respectively) compared to the pTEE method (7.2 and 12.0% in men and women, respectively) [23]. Both the American and the Spanish studies reported lower proportions of EI-MR using both the Goldberg and the pTEE methods compared to the current study. These different proportions of misreporters identified in previous literature could be due to methodological differences such as different tools for collecting dietary intake data, differences in BMR equations, differences in the categorization of PAL, or cut-off equations.

Despite differences in methodology, other studies have used Inclusion and ExBefore scenarios to address misestimation of EI and its impacts on dietary patterns [44–48]. A Swedish population-based study found that the composition of two dietary patterns differed with intakes of coffee and tea between Inclusion and ExBefore scenarios [44]. A cross-sectional study of Norwegian women likewise reported that the food group composition of dietary patterns differed between Inclusion and ExBefore scenarios [48].

A participant’s dietary pattern assignment could have been different across methods of identifying EI-MR and also across the four scenarios to account for EI-MR due to the arbitrary nature of k-means cluster analysis. This may explain why the differing scenarios to account for EI-MR appear to impact the results of the cluster analysis and hence the composition of each dietary pattern. While steps were taken to limit subjectivity for *k*-means cluster analysis, some decisions are reliant on researchers’ perspective and intuition in terms of what is the most “meaningful” cluster solution. Despite this limitation, *k*-means cluster analysis has been shown to be the optimal method to ensure cluster reproducibility [9, 10]. Total rEI was selected as the optimal input variable for the *k*-means cluster analysis because it is considered a surrogate measure for total food consumed and EI is the foundation of the diet [16]. Other studies have used different measures such as daily intake frequencies [49] and the average weight of food consumed per day [50] to define clusters that may impact the results of the cluster analysis and hence, the composition of the dietary pattern. There is no agreed-upon approach for addressing error in dietary intake assessment. In the current study we applied four different scenarios to address this issue, using EI as an approximation for overall diet. Each scenario led to different findings, indicating that the approach for addressing error in dietary intake might have implications for the comparability of studies and the ability to make recommendations for policy and practice. Given that there is no marker of true usual dietary patterns, it is not possible to ascertain which method or scenario for identifying EI misestimation results in the most accurate dietary patterns. Nonetheless, this study does highlight that the methods and scenarios used can impact the results.

The findings of this study should be interpreted in light of several considerations. The large sample size (> 25, 000) is a strength, providing more accurate mean values and smaller error margins [51]. We were also able to use a more comprehensive measure of physical activity which takes into account the frequency, intensity and duration of several domains of activity [29], as opposed to a similar study that used intensity and duration, but did not capture frequency [24]. Yet there are several limitations; first, the FFQ was a closed-ended survey with a limited list of food and beverage items, and a limited range of frequency and portion size options that can be reported. Further, FFQ data have been demonstrated to be affected by systematic measurement error to a greater extent than data collected from 24-h recalls and food records [52]. The use of these short-term tools in cohort studies may be helpful to mitigate the impact of measurement errors [53] by providing more comprehensive information on eating patterns and food combinations [12]. Measurement error also impacts estimates of physical activity, which were used in calculations of EI misreporting [54]. This might have influenced the assignment of PAL for some participants.

## Conclusion

We observed that the pTEE method identified a significantly higher proportion of EI-MRs compared to the revised-Goldberg method. Different scenarios used to account for EI-MR appear to influence the composition of the dietary pattern. Continued efforts are needed to explore and validate methods and their ability to identify and mitigate the impact of EI misestimation in nutritional epidemiology. The use of biomarkers such as DLW in a subset of participants within a cohort study, and other validation methods, can improve knowledge of misreporting and optimal strategies to minimize the bias that might occur when analyzing dietary data for a given context and population. Furthermore, sensitivity testing should be conducted alongside additional work to improve dietary assessment methods and correction approaches for dietary intake misestimation, such that valid conclusions about the relationship between dietary intake and health outcomes can be drawn.

## Supplementary Information

**Additional file 1.**

**Additional file 2.**

## Data Availability

The data that support the findings of this study are available from Alberta’s Tomorrow Project, but restrictions apply to the availability of these data, which were used under license for the current study, and so are not publicly available. Data are however available from Alberta’s Tomorrow Project Research (www.myatpresearch.ca) upon reasonable request.

## Abbreviations

BMR: Basal Metabolic Rate
CDHQ-I: Canadian Diet History Questionnaire, version 1
DLW: Doubly Labeled Water
FFQ: Food Frequency Questionnaire
EE: Energy Expenditure
EI: Energy Intake
EI-MR: Energy Intake Misreporters
EI-OR: Energy Intake Over-Reporters
EI-UR: Energy Intake Under-Reporters
EI-PR: Energy Intake Plausible Reporters
ExBefore: Energy intake misreporters are excluded prior to completing the cluster analysis
ExAfter: Energy intake misreporters are excluded after completing the cluster analysis
HLQ: Health and Lifestyle Questionnaire
Inclusion: Energy intake misreporters are included in the cluster analysis
InclusionNN: Energy intake misreporters are excluded before the cluster analysis but added to the ExBefore cluster solution using the nearest neighbor method
PAL: Physical Activity Level
pTEE: Predicted Total Energy Expenditure
PYTPAQ: Past-Year Total Physical Activity Questionnaire
rEI: Reported Energy Intake

## References

[1] Hu FB (2002). Dietary pattern analysis: a new direction in nutritional epidemiology. Curr Opin Lipidol.

[2] Reedy J, Subar AF, George SM, Krebs-Smith SM (2018). Extending methods in dietary patterns research. Nutrients..

[3] Hoffmann I. Transcending reductionism in nutrition research. Am J Clin Nutr. 2003;78(3 Suppl):514S–516S. 10.1093/ajcn/78.3.514S 12936942

[4] Jacobs DR, Tapsell LC (2007). Food, not nutrients, is the fundamental unit in nutrition. Nutr Rev.

[5] Bodnar LM, Cartus AR, Kirkpatrick SI, Himes KP, Kennedy EH, Simhan HN, Grobman WA, Duffy JY, Silver RM, Parry S, Naimi AI (2020). Machine learning as a strategy to account for dietary synergy: an illustration based on dietary intake and adverse pregnancy outcomes. Am J Clin Nutr.

[6] World Cancer Research Fund/American Institute for Cancer Research. Diet, Nutrition, Physical Activity and Cancer: a Global Perspective. Continuous Update Project Expert Report 2018. 2018. 1–53 p. Available from: http://gco.iarc.fr/today%0Adietandcancerreport.org

[7] Grosso G, Bella F, Godos J, Sciacca S, Del Rio D, Ray S (2017). Possible role of diet in cancer: systematic review and multiple meta-analyses of dietary patterns, lifestyle factors, and cancer risk. Nutr Rev.

[8] Newby PK, Tucker KL (2004). Empirically derived eating patterns using factor or cluster analysis: a review. Nutr Rev.

[9] Sauvageot N, Schritz A, Leite S, Alkerwi A, Stranges S, Zannad F (2017). Stability-based validation of dietary patterns obtained by cluster analysis. Nutr J.

[10] Lo Siou G, Yasui Y, Csizmadi I, McGregor SE, Robson PJ (2011). Exploring statistical approaches to diminish subjectivity of cluster analysis to derive dietary patterns: the tomorrow project. Am J Epidemiol.

[11] Illner A-K, Freisling H, Boeing H, Huybrechts I, Crispim SP, Slimani N (2012). Review and evaluation of innovative technologies for measuring diet in nutritional epidemiology. Int J Epidemiol.

[12] Subar AF, Freedman LS, Tooze JA, Kirkpatrick SI, Boushey C, Neuhouser ML, Thompson FE, Potischman N, Guenther PM, Tarasuk V, Reedy J, Krebs-Smith SM (2015). Addressing current criticism regarding the value of self-report dietary data. J Nutr.

[13] Devlin UM, Mcnulty BA, Nugent AP, Gibney MJ (2012). The use of cluster analysis to derive dietary patterns: methodological considerations, reproducibility, validity and the effect of energy mis-reporting. Proc Nutr Soc.

[14] Gomes D, Luque V, Xhonneux A, Verduci E, Socha P, Koletzko B, Berger U, Grote V (2018). A simple method for identification of misreporting of energy intake from infancy to school age: results from a longitudinal study. Clin Nutr.

[15] Solbak NM, Al Rajabi A, Akawung AK, Lo Siou G, Kirkpatrick SI, Robson PJ (2019). Strategies to address misestimation of energy intake based on self-report dietary consumption in examining associations between dietary patterns and cancer risk. Nutrients..

[16] Livingstone MBE, Black AEE (2003). Markers of the validity of reported energy intake. J Nutr.

[17] Banna JC, McCrory MA, Fialkowski MK, Boushey C (2017). Examining plausibility of self-reported energy intake data: considerations for method selection. Front Nutr.

[18] Black AE (2000). Critical evaluation of energy intake using the Goldberg cut-off for energy intake:basal metabolic rate. A practical guide to its calculation, use and limitations. Int J Obes Relat Metab Disord.

[19] McCrory MA, Hajduk CL, Roberts SB (2002). Procedures for screening out inaccurate reports of dietary energy intake. Public Health Nutr.

[20] Huang TT-K, Roberts SB, Howarth NC, McCrory MA (2005). Effect of screening out implausible energy intake reports on relationships between diet and BMI. Obes Res.

[21] Tooze JA, Krebs-Smith SM, Troiano RP, Subar AF (2012). The accuracy of the Goldberg method for classifying misreporters of energy intake on a food frequency questionnaire and 24-h recalls: comparison with doubly labeled water. Eur J Clin Nutr.

[22] Jessri M, Lou WY, L’Abbé MR (2016). Evaluation of different methods to handle misreporting in obesity research: evidence from the Canadian national nutrition survey. Br J Nutr.

[23] Mendez MA, Popkin BM, Buckland G, Schroder H, Amiano P (2011). Alternative methods of accounting for underreporting and Overreporting when measuring dietary intake-obesity relations. Am J Epidemiol.

[24] Rhee JJ, Sampson L, Cho E, Hughes MD, Hu FB, Willett WC (2015). Comparison of methods to account for implausible reporting of energy intake in epidemiologic studies. Am J Epidemiol.

[25] Robson PJ, Solbak NM, Haig TR, Whelan HK, Vena JE, Akawung AK, Rosner WK, Brenner DR, Cook LS, Csizmadi I, Kopciuk KA, McGregor SE, Friedenreich CM (2016). Design, methods and demographics from phase I of Alberta’s tomorrow project cohort: a prospective cohort profile. C open.

[26] Ye M, Robson PJ, Eurich DT, Vena JE, Xu J-Y, Johnson JA (2017). Cohort Profile: Alberta’s Tomorrow Project. Int J Epidemiol.

[27] Bryant H, Robson PJ, Ullman R, Friedenreich C, Dawe U (2006). Population-based cohort development in Alberta, Canada: a feasibility study. Chronic Dis Can.

[28] Csizmadi I, Kahle L, Ullman R, Dawe U, Zimmerman TP, Friedenreich CM, Bryant H, Subar AF (2007). Adaptation and evaluation of the National Cancer Institute’s diet history questionnaire and nutrient database for Canadian populations. Public Health Nutr.

[29] Friedenreich CM, Courneya KS, Neilson HK, Matthews CE, Willis G, Irwin M, Troiano R, Ballard-Barbash R (2006). Reliability and validity of the past year Total physical activity questionnaire. Am J Epidemiol.

[30] National Cancer Institute. Diet History Questionnaire: Canadian Version. 2005. Available from: https://epi.grants.cancer.gov/dhq/forms/canadian/

[31] Csizmadi I, Lo Siou G, Friedenreich CM, Owen N, Robson PJ (2011). Hours spent and energy expended in physical activity domains: results from the tomorrow project cohort in Alberta, Canada. Int J Behav Nutr Phys Act.

[32] Mifflin MD, St Jeor ST, Hill LA, Scott BJ, Daugherty SA, Koh YO (1990). A new predictive equation for resting energy expenditure in healthy individuals. Am J Clin Nutr.

[33] Goldberg GR, Black AE, Jebb SA, Cole TJ, Murgatroyd PR, Coward WA, Prentice AM (1991). Critical evaluation of energy intake data using fundamental principles of energy physiology: 1. Derivation of cut-off limits to identify under-recording. Eur J Clin Nutr.

[34] Brooks GA, Butte NF, Rand WM, Flatt J-P, Caballero B (2004). Chronicle of the Institute of medicine physical activity recommendation: how a physical activity recommendation came to be among dietary recommendations. Am J Clin Nutr.

[35] Amirkalali B, Najafi M, Ataie-Jafari A, Hosseini S, Heshmat R (2008). Under- and overreporting of energy in a group of candidates for CABG surgery and its association with some anthropometric and sociodemographic factors , Tehran, Iran. Vasc Health Risk Manag.

[36] Food and Nutrition Board (2005). Institute of Medicine. Dietary Reference Intakes for Energy, Carbohydrate, Fiber, Fat, Fatty acids, Cholesterol, Protein and Amino Acids.

[37] Black AE, Cole TJ (2000). Within- and between-subject variation in energy expenditure measured by the doubly-labelled water technique: implications for validating reported dietary energy intake. Eur J Clin Nutr.

[38] Garriguet D (2008). Impact of identifying plausible respondents on the under-reporting of energy intake in the Canadian community health survey. Heal reports.

[39] Forgy E (1965). Cluster analysis of multivariate data : efficiency versus interpretability of classifications. Biometrics..

[40] Hu L-Y, Huang M-W, Ke S-W, Tsai C-F (2016). The distance function effect on k-nearest neighbor classification for medical datasets. Springerplus..

[41] Cooper MC, Milligan GW (1988). A study of standardization of variables in cluster analysis. J Classif.

[42] Hubert L, Arabie P (1985). Comparing partitions. J Classif.

[43] Landis JR, Koch GG (1977). The measurement of observer agreement for categorical data. Biometrics..

[44] Winkvist A, Hörnell A, Hallmans G, Lindahl B, Weinehall L, Johansson I (2009). More distinct food intake patterns among women than men in northern Sweden: a population-based survey. Nutr J.

[45] Martikainen P, Brunner E, Marmot M (2003). Socioeconomic differences in dietary patterns among middle-aged men and women. Soc Sci Med.

[46] Bailey RL, Mitchell DC, Miller C, Smiciklas-wright H (2007). Assessing the effect of underreporting energy intake on dietary patterns and weight status. J Am Diet Assoc.

[47] Funtikova AN, Gomez SF, Fitó M, Elosua R, Benítez-Arciniega AA, Schröder H (2015). Effect of energy under-reporting on secular trends of dietary patterns in a mediterranean population. PLoS One.

[48] Markussen MS, Veierød MB, Ursin G, Andersen LF (2016). The effect of under-reporting of energy intake on dietary patterns and on the associations between dietary patterns and self-reported chronic disease in women aged 50-69 years. Br J Nutr.

[49] Thorpe MG, Milte CM, Crawford D, McNaughton SA (2016). A comparison of the dietary patterns derived by principal component analysis and cluster analysis in older Australians. Int J Behav Nutr Phys Act.

[50] Pérez-Rodrigo C, Gil Á, González-Gross M, Ortega RM, Serra-Majem L, Varela-Moreiras G, Aranceta-Bartrina J (2015). Clustering of dietary patterns, lifestyles, and overweight among Spanish children and adolescents in the ANIBES study. Nutrients..

[51] Biau DJ, Kernéis S, Porcher R (2008). Statistics in brief: the importance of sample size in the planning and interpretation of medical research. Clin Orthop Relat Res.

[52] Freedman LS, Commins JM, Moler JE, Arab L, Baer DJ, Kipnis V, Midthune D, Moshfegh AJ, Neuhouser ML, Prentice RL, Schatzkin A, Spiegelman D, Subar AF, Tinker LF, Willett W (2014). Pooled results from 5 validation studies of dietary self-report instruments using recovery biomarkers for energy and protein intake. Am J Epidemiol.

[53] Carroll RJ, Midthune D, Subar AF, Shumakovich M, Freedman LS, Thompson FE (2012). Practice of epidemiology taking advantage of the strengths of 2 different dietary assessment instruments to improve intake estimates for nutritional epidemiology. Pract Epidemiol.

[54] Tooze JA, Troiano RP, Carroll RJ, Moshfegh AJ, Freedman LS (2013). A measurement error model for physical activity level as measured by a questionnaire with application to the 1999-2006 NHANES questionnaire. Am J Epidemiol.

