# Supplementary material for: The effect of different methods to identify, and scenarios used to address energy intake misestimation on dietary patterns derived by cluster analysis
Source: Nutr J. 2021 May 8;20:42. doi: 10.1186/s12937-021-00696-3 (PMC8106845; doi:10.1186/s12937-021-00696-3)
Supplement: Supplementary file 1 — Additional file 1. [file 12937_2021_696_MOESM1_ESM.docx]

**Cluster Labels (in descending order of mean intake)**

1. **Men with misreporters included (Inclusion)**
2. **Two cluster solution**
3. **Cluster 1**: meat, pasta and pizza, beer, soda regular, other bread, processed meat, chips, jam, cake, cooked potatoes, cheese regular, eggs, French fries, confectionery, dessert, liquor, ice cream, margarine, salad dressing regular, high fat dairy, butter, coffee
4. **Cluster 2**: fruits, low fat dairy, breakfast cereal, whole meal bread, fruit juice, rice, poultry, nuts, dairy regular, soup, cooked vegetables, fish, wine, legumes
5. **Three cluster solution**
6. **Cluster 1:** fruits, breakfast cereal, fruit juice, rice, nuts, poultry (no skin), dairy regular, cooked vegetables, soup, fish, wine, legumes meal replacement
7. **Cluster 2:** meat, pasta and pizza, beer, soda regular, chips, other bread, processed meat, cheese regular, French fries, eggs, liquor, salad dressing regular, butter, high fat dairy
8. **Cluster 3:** low fat dairy, whole meal bread, jam, cake, cooked potatoes, dessert, confectionery, margarine, ice cream, coffee
9. **Four cluster solution**
10. **Cluster 1:** meat, processed meat, other bread, French fries, butter, fish
11. **Cluster 2:** pasta and pizza, beer, low fat dairy, soda regular, chips, cheese regular, confectionery, liquor, ice cream, high fat dairy, wine
12. **Cluster 3:** fruits, breakfast cereal, fruit juice, rice, nuts, poultry, dairy regular, cooked vegetables, soup, fish, wine, legumes
13. **Cluster 4:** whole meal bread, jam, cake, cooked potatoes, other bread, margarine, dessert, ice cream, coffee, high fat dairy
14. **Five cluster solution**
15. **Cluster 1:** whole meal bread, jam, cake, cooked potatoes, other bread, margarine, coffee, high fat dairy
16. **Cluster 2:** meat, processed meat, other bread, eggs, French fries, salad dressing regular, butter, Mexican
17. **Cluster 3:** fruits, breakfast cereal, fruit juice, rice, nuts, poultry, regular dairy, cooked vegetables, soup, fish, wine, legumes, meal replacement
18. **Cluster 4:** beer, wine
19. **Cluster 5:** pasta and pizza, low fat dairy, soda regular, chips, other bread, cheese regular, confectionery, dessert, liquor, Mexican
20. **Six cluster solution**
21. **Cluster 1:** meat, processed meat, other bread, eggs, butter, salad dressing regular, soup, cooked vegetables,
22. **Cluster 2:** whole meal bread, cake, cooked potatoes, margarine, dessert, ice cream, coffee, high fat dairy
23. **Cluster 3:** low fat dairy, breakfast cereal, fruit juice
24. **Cluster 4:** pasta and pizza, soda regular, chips, cheese regular, confectionery, French fries, dessert, liquor, ice cream, soup, high fat dairy
25. **Cluster 5:** beer
26. **Cluster 6:** fruits, fruit juice, rice, poultry, dairy regular, soup, fish, wine
27. **Seven cluster solution**
28. **Cluster 1:** butter, other bread, eggs, salad dressing regular, liquor, high fat dairy
29. **Cluster 2:** whole meal bread, jam, cooked potatoes, margarine, coffee
30. **Cluster 3:** meat, pasta and pizza, soda regular, chips, processed meat, French fries, poultry, liquor
31. **Cluster 4:** beer, cake
32. **Cluster 5:** low fat dairy, breakfast cereal, dessert, ice cream
33. **Cluster 6:** fruit, fruit juice, rice, nuts, poultry, regular dairy, cooked vegetables, fish, wine
34. **Cluster 7:** cheese regular, chips, confectionery
35. **Men with misreporters excluded (revised-Goldberg)**
36. **Two cluster solution**
37. **Cluster 1**: meat, pasta and pizza, beer, soda regular, jam, chips, processed meat, other bread, cake, cooked potatoes, cheese regular, French fries, confectionery, eggs, dessert, margarine, ice cream, liquor, salad dressing regular, high fat dairy, coffee, butter
38. **Cluster 2**: fruits, low fat dairy, fruit juice, breakfast cereal, whole meal bread, rice, nuts, poultry, dairy regular, soup, cooked vegetables, fish, wine, legumes

**b1. Three cluster solution (misreporters excluded BEFORE cluster analysis) (ExBefore)**

1. **Cluster 1:** fruits, low fat dairy, fruit juice, breakfast cereal, rice, nuts, poultry, dairy regular, soup, cooked vegetables, fish, wine, meal replacement, legumes, olive oil
2. **Cluster 2:** meat, pasta and pizza, beer, soda regular, chips, processed meat, cheese regular, French fries, confectionery, liquor, salad dressing regular, Mexican, butter
3. **Cluster 3:** jam, whole meal bread, cake, other bread, cooked potatoes, margarine, eggs, dessert, coffee, ice cream, high fat dairy

**b2. Three cluster solution (misreporters excluded AFTER cluster analysis) (ExAfter)**

1. **Cluster 1:** fruits, fruit juice, breakfast cereal, rice, nuts, poultry (no skin), dairy regular, cooked vegetables, soup, fish, wine, meal replacement, legumes
2. **Cluster 2:** meat, pasta and pizza, beer, soda regular, chips, other bread, processed meat, cheese regular, French fries, eggs, liquor, salad dressing, butter, high fat dairy, Mexican
3. **Cluster 3:** low fat dairy, cake, whole meal bread, jam, cooked potatoes, confectionery, dessert, ice cream, margarine, coffee
4. **Four cluster solution**
5. **Cluster 1:** fruit, low fat dairy, fruit juice, breakfast cereal, rice, nuts, poultry, regular dairy, cooked vegetables, soup, fish, wine, legumes
6. **Cluster 2:** whole meal bread, jam, margarine, cake, cooked potatoes, other bread, dessert
7. **Cluster 3:** meat, pasta and pizza, beer, soda regular, chips, processed meat, other bread, French fries, liquor
8. **Cluster 4:** jam, cheese regular, confectionery, eggs, coffee, dessert, high fat dairy, ice cream, butter, salad dressing regular
9. **Five cluster solution**
10. **Cluster 1:** meat, pasta and pizza, beer, processed meat, liquor, salad dressing regular, butter
11. **Cluster 2:** jam, cheese regular, coffee, eggs, high fat dairy, dessert, ice cream, soup
12. **Cluster 3:** whole meal bread, margarine, cake, other bread, cooked potatoes, processed meat, dessert
13. **Cluster 4:** chips, pasta and pizza, soda regular, cheese regular, confectionery, French fries, ice cream
14. **Cluster 5:** fruit, low fat dairy, fruit juice, breakfast cereal, nuts, rice, poultry, regular dairy, cooked vegetables, soup fish, wine, meal replacement
15. **Six cluster solution**
16. **Cluster 1:** whole meal bread, margarine, cake, other bread, cooked potatoes, dessert
17. **Cluster 2:** jam, cheese regular, high fat dairy, dessert, ice cream, soup, butter
18. **Cluster 3:** beer
19. **Cluster 4:** fruit, low fat dairy, fruit juice, breakfast cereal, rice, nuts, poultry, cooked vegetables, fish, eggs, meal replacement
20. **Cluster 5:** chips, pasta and pizza, soda regular, confectionery, French fries, ice cream, butter
21. **Cluster 6:** meat, processed meat, eggs, liquor, dessert, salad dressing regular
22. **Seven cluster solution**
23. **Cluster 1:** meat, processed meat, cheese regular, eggs, liquor, salad dressing regular, butter
24. **Cluster 2:** low fat dairy, fruit juice, cake, breakfast cereal, dessert, ice cream
25. **Cluster 3:** chips, pasta and pizza, soda regular, cheese regular, confectionery, French fries
26. **Cluster 4:** whole meal bread, margarine, other bread, cooked potatoes
27. **Cluster 5:** jam, cheese regular, high fat dairy, dessert, ice cream
28. **Cluster 6:** beer
29. **Cluster 7:** rice, nuts, poultry, regular dairy, cooked vegetables, soup, fish, wine, meal replacement
30. **Men with misreporters excluded (pTEE)**
31. **Two cluster solution**
32. **Cluster 1**: low fat dairy, fruit juice, whole meal bread, breakfast cereal, rice, nuts, poultry, dairy regular, soup, cooked vegetables, fish, wine, legumes, meal replacement
33. **Cluster 2**: meat, pasta and pizza, beer, soda regular, chips, jam, other bread, processed meat, cake, cooked potatoes, cheese regular, confectionery, French fries, eggs, dessert, margarine, ice cream, liquor, salad dressing regular, high fat dairy, butter, coffee

**b1. Three cluster solution (misreporters excluded BEFORE cluster analysis) (ExBefore)**

1. **Cluster 1:** meat, pasta and pizza, beer, soda regular, chips, processed meat, cheese regular, confectionery, French fries, liquor, salad dressing regular, butter, Mexican
2. **Cluster 2:** jam, whole meal bread, cake, other bread, cooked potatoes, margarine, eggs, dessert, coffee, ice cream, high fat dairy
3. **Cluster 3:** fruits, low fat dairy, fruit juice, breakfast cereal, nuts, rice, poultry (no skin), dairy regular, cooked vegetables, soup, fish, wine, meal replacement, legumes

**b2. Three cluster solution (misreporters excluded AFTER cluster analysis) (ExAfter)**

1. **Cluster 1:** fruits, fruit juice, breakfast cereal, rice, nuts, poultry (no skin), dairy regular, cooked vegetables, soup, meal replacement, fish, wine, legumes, Mexican, olive oil
2. **Cluster 2:** meat, pasta and pizza, beer, soda regular, chips, other bread, processed meat, cheese regular, French fries, eggs, liquor, salad dressing regular, butter, high fat dairy
3. **Cluster 3:** low fat dairy, cake, whole meal bread, jam, cooked potatoes, confectionery, dessert, ice cream, margarine, coffee
4. **Four cluster solution**
5. **Cluster 1:** meat, pasta and pizza, beer, soda regular, chips, processed meat, French fries, liquor
6. **Cluster 2:** whole meal bread, cake, cooked potatoes, other bread, eggs, dessert, salad dressing regular
7. **Cluster 3:** fruits, low fat dairy, fruit juice, breakfast cereal, nuts, rice, poultry, dairy regular, cooked vegetables, soup, fish, wine, meal replacement
8. **Cluster 4:** jam, cheese regular, confectionery, coffee, eggs, high fat dairy, ice cream, soup, salad dressing regular, butter
9. **Five cluster solution**
10. **Cluster 1:** jam, cheese regular, coffee, high fat dairy, ice cream, soup
11. **Cluster 2:** meat, beer, processed meat, liquor, salad dressing regular, butter
12. **Cluster 3:** fruit, low fat dairy, fruit juice, breakfast cereal, nuts, rice, poultry, dairy regular, cooked vegetables, soup, fish, wine, legumes
13. **Cluster 4:** whole meal bread, margarine, cake, other bread, cooked potatoes, dessert, eggs
14. **Cluster 5:** chips, pasta and pizza, soda regular, cheese regular, French fries, confectionery, ice cream
15. **Six cluster solution**
16. **Cluster 1:** jam, cheese regular, coffee, high fat dairy, ice cream
17. **Cluster 2:** chips, pasta and pizza, soda regular, French fries, confectionery, liquor
18. **Cluster 3:** beer, wine
19. **Cluster 4:** whole meal bread, cake, cooked potatoes, other bread, dessert
20. **Cluster 5:** fruit, low fat dairy, fruit juice, breakfast cereal, rice, poultry, cooked vegetables, soup, fish, wine
21. **Cluster 6:** meat, processed meat, eggs, liquor, salad dressing regular, butter
22. **Seven cluster solution**
23. **Cluster 1:** fruit, rice, nuts, dairy regular, poultry, cooked vegetables, soup, meal replacement, fish, wine, legumes
24. **Cluster 2:** whole meal bread, margarine, cooked potatoes, other bread
25. **Cluster 3:** jam, cheese regular, coffee, high fat dairy, ice cream
26. **Cluster 4:** beer
27. **Cluster 5:** low fat dairy, fruit juice, cake, breakfast cereal, dessert
28. **Cluster 6:** chips, pasta and pizza, soda regular, French fries, confectionery
29. **Cluster 7:** meat, processed meat, eggs, liquor, salad dressing regular, butter
