# Supplementary material for: The effect of different methods to identify, and scenarios used to address energy intake misestimation on dietary patterns derived by cluster analysis
Source: Nutr J. 2021 May 8;20:42. doi: 10.1186/s12937-021-00696-3 (PMC8106845; doi:10.1186/s12937-021-00696-3)
Supplement: Supplementary file 2 — Additional file 2. [file 12937_2021_696_MOESM2_ESM.docx]

**Cluster Labels (in descending order of mean intake)**

1. **Women with misreporters included (Inclusion)**
2. **Two cluster solution**
3. **Cluster 1**: Fruit, low fat dairy, regular diary, breakfast cereal, poultry, fruit juice, nuts, whole meal bread, rice, cooked vegetables, soup, fish, wine, legumes, raw vegetables, cabbage
4. **Cluster 2**: meat, pasta and pizza, chips, cake, soda regular, other bread cooked potatoes, jam, cheese regular, confectionery, processed meat, eggs, salad dressing regular, dessert, margarine, butter, French fries, Mexican, coffee, ice cream, beer
5. **Three cluster solution**
6. **Cluster 1:** fruits, dairy regular, poultry (no skin), nuts, rice, cooked vegetables, fish, soup, wine, legumes, raw vegetables, cabbage, meal replacement
7. **Cluster 2:** meat, pasta and pizza, chips, soda regular, other bread, cooked potatoes, cheese regular, processed meat, confectionery, eggs, salad dressing regular, dessert, margarine, butter, French fries, Mexican, beer, coffee, high fat dairy, liquor
8. **Cluster 3:** low fat dairy, breakfast cereal, whole meal bread, fruit juice, cake, jam, ice cream
9. **Four cluster solution**
10. **Cluster 1:** meat, pasta and pizza, chips, soda regular, cheese regular, confectionery, processed meat, eggs, salad dressing regular, wine, Mexican, French fries, beer, liquor, high fat dairy
11. **Cluster 2:** low fat dairy, breakfast cereal, fruit juice
12. **Cluster 3:** fruits, dairy regular, poultry, nuts, rice, cooked vegetables, fish, legumes, raw vegetables, cabbage, meal replacement
13. **Cluster 4:** whole meal bread, cooked potatoes, cake, other bread, jam, margarine, soup, butter, dessert, coffee, ice cream, mayonnaise
14. **Five cluster solution**
15. **Cluster 1:** Poultry, rice, eggs, fish, wine, meal replacement, olive oil
16. **Cluster 2:** meat, pasta and pizza, chips, soda regular, cheese regular, confectionery, processed meat, salad dressing regular, French fries, beer, Mexican, liquor, high fata dairy
17. **Cluster 3:** fruits, dairy regular, nuts, cooked vegetables, soup, legumes, raw vegetables, cabbage
18. **Cluster 4:** whole meal bread, cooked potatoes, cake, jam, other bread, margarine, butter, dessert, coffee, ice cream, mayonnaise
19. **Cluster 5:** low fat dairy, breakfast cereal, fruit juice
20. **Six cluster solution**
21. **Cluster 1:** low fat dairy, breakfast cereal, fruit juice
22. **Cluster 2:** poultry, rice, eggs, fish, meal replacement, olive oil
23. **Cluster 3:** pasta and pizza, chips, soda regular, cheese regular, confectionery, wine, dessert, beer, Mexican, liquor, high fat dairy
24. **Cluster 4:** fruits, dairy regular, nuts, cooked vegetables, soup, legumes, raw vegetables, cabbage
25. **Cluster 5:** whole meal bread, jam, cake, mayonnaise
26. **Cluster 6:** meat, cooked potatoes, other bread, processed meat, margarine, salad dressing, butter, coffee
27. **Seven cluster solution**
28. **Cluster 1:** fruit, rice, cooked vegetables, soup, legumes, raw vegetables, cabbage
29. **Cluster 2:** pasta and pizza, chips, soda regular, cheese regular, confectionery, dessert, beer, Mexican, French fries, liquor, high fat dairy
30. **Cluster 3:** low fat dairy, breakfast cereal, fruit juice
31. **Cluster 4:** dairy regular, nuts
32. **Cluster 5:** whole meal bread, jam, cake, butter, mayonnaise
33. **Cluster 6:** poultry, eggs, fish, meal replacement, olive oil
34. **Cluster 7:** meat, cooked potatoes, other bread, processed meat, margarine, salad dressing regular, coffee, ice cream
35. **Women with misreporters excluded (revised-Goldberg)**
36. **Two cluster solution**
37. **Cluster 1**: fruits, low fat dairy, breakfast cereal, regular dairy, poultry, nuts, fruit juice, whole meal bread, rice, cooked vegetables, soup, fish, wine, legumes, raw vegetables, cabbage
38. **Cluster 2**: meat, pasta and pizza, chips, soda regular, cake, other bread, jam, cheese regular, cooked potatoes, confectionery, processed meat, eggs, salad dressing regular, dessert, margarine, butter, French fries, ice cream, Mexican, beer, high fat dairy, coffee

**b1. Three cluster solution (misreporters excluded BEFORE cluster analysis) (ExBefore)**

1. **Cluster 1:** fruits, dairy regular, poultry (no skin), nuts, whole meal bread, rice, cooked vegetables, soup, fish, wine, legumes, raw vegetables, cabbage, meal replacement, olive oil
2. **Cluster 2:** meat, pasta and pizza, chips, soda regular, cake, other bread, jam, cheese regular, cooked potatoes, confectionery, processed meat, eggs, salad dressing regular, dessert, margarine, butter, French fries, beer, ice cream, Mexican, high fat dairy, liquor, coffee
3. **Cluster 3:** low fat dairy, breakfast cereal, fruit juice

**b2. Three cluster solution (misreporters excluded AFTER cluster analysis) (ExAfter)**

1. **Cluster 1:** fruit, dairy regular, poultry (no skin), nuts, rice, cooked vegetables, fish, soup, wine, legumes, raw vegetables, cabbage, meal replacement, olive oil
2. **Cluster 2:** meat, pasta and pizza, chips, soda regular, other bread, cooked potatoes, cheese regular, confectionery, processed meat, salad dressing regular, eggs, dessert, margarine, butter, French fries, Mexican, beer, high fat dairy, liquor
3. **Cluster 3:** low fat dairy, breakfast cereal, whole meal bread, fruit juice, cake, jam, ice cream
4. **Four cluster solution**
5. **Cluster 1:** low fat dairy, breakfast cereal
6. **Cluster 2:** meat, pasta and pizza, chips, soda regular, other bread, jam, confectionery, cheese regular, processed meat, salad dressing regular, dessert, margarine, butter, French fries, beer, ice cream, Mexican, high fat dairy, liquor, coffee
7. **Cluster 3:** fruits, poultry, dairy regular, nuts, fruit juice, rice, cooked vegetables, fish, legumes, raw vegetables, cabbage, meal replacement
8. **Cluster 4:** whole meal bread, cake, cooked potatoes, eggs, soup, wine, hot tea
9. **Five cluster solution**
10. **Cluster 1:** whole meal bread, soup, wine, hot tea
11. **Cluster 2:** low fat dairy, breakfast cereal,
12. **Cluster 3:** cake, jam, cooked potatoes, other bread, cheese regular, confectionery, processed meat, eggs, salad dressing, margarine, butter, dessert, high fat dairy, coffee, beer, ice cream, liquor, mayonnaise
13. **Cluster 4:** fruit, poultry, dairy regular, nuts, fruit juice, rice, cooked vegetables, fish, legumes, raw vegetables, cabbage, meal replacement, olive oil
14. **Cluster 5:** pasta and pizza, meat, chips, soda regular, French fries, Mexican
15. **Six cluster solution**
16. **Cluster 1:** poultry, rice, fish, wine, meal replacement, olive oil
17. **Cluster 2:** fruits, dairy regular, nuts, fruit juice, cooked vegetables, legumes, raw vegetables, cabbage
18. **Cluster 3:** cake, cooked potatoes, jam, other bread, cheese regular, confectionery, eggs, salad dressing regular, butter, margarine, dessert, high fat dairy, beer, coffee, ice cream, liquor, mayonnaise
19. **Cluster 4:** pasta and pizza, meat, chips, soda regular, French fries, Mexican
20. **Cluster 5:** whole meal bread, soup, hot tea
21. **Cluster 6:** low fat dairy, breakfast cereal
22. **Seven cluster solution**
23. **Cluster 1:** soup, hot tea
24. **Cluster 2:** meat, soda regular, chips, confectionery, other bread, cheese regular, processed meat, salad dressing regular, butter, French fries, beer, liquor, high fat dairy
25. **Cluster 3:** pasta and pizza, Mexican
26. **Cluster 4:** poultry, rice, fish, wine, meal replacement, olive oil
27. **Cluster 5:** low fat dairy, breakfast cereal
28. **Cluster 6:** whole meal bread, jam, cake, cooked potatoes, margarine, eggs, dessert, coffee, ice cream, mayonnaise
29. **Cluster 7:** fruits, dairy regular, nuts, fruit juice, cooked vegetables, legumes, cabbage
30. **Women with misreporters excluded (pTEE)**
31. **Two cluster solution**
32. **Cluster 1**: meat, pasta and pizza, chips, soda regular, cake, other bread, cheese regular, jam, cooked potatoes, confectionery, processed meat, salad dressing regular, eggs, dessert, margarine, butter, wine, French fries, ice cream, beer, Mexican, high fat dairy, coffee
33. **Cluster 2**: fruits, low fat dairy, breakfast cereal, fruit juice, dairy regular, poultry, nuts, whole meal bread, rice, cooked vegetables, soup, fish, legumes, raw vegetables, cabbage

**b1. Three cluster solution (misreporters excluded BEFORE cluster analysis) (ExBefore)**

1. **Cluster 1:** meat, pasta and pizza, chips, soda regular, other bread, cheese regular, jam, cooked potatoes, confectionery, processed meat, salad dressing regular, eggs, dessert, margarine, butter, French fries, beer, Mexican, high fat dairy, liquor, coffee
2. **Cluster 2:** fruits, dairy regular, nuts, poultry (no skin), rice, cooked vegetables, fish, soup, wine, legumes, raw vegetables, cabbage, meal replacement, olive oil
3. **Cluster 3:** low fat dairy, breakfast cereal, fruit juice, whole meal bread, cake, ice cream

**b2. Three cluster solution (misreporters excluded AFTER cluster analysis) (ExAfter)**

1. **Cluster 1:** fruit, dairy regular, poultry, nuts, rice, cooked vegetables, fish, soup, wine, legumes, raw vegetables, cabbage, meal replacement, olive oil
2. **Cluster 2:** meat, pasta and pizza, chips, soda regular, other bread, cheese regular, cooked potatoes, confectionery, processed meat, salad dressing regular, eggs, dessert, margarine, butter, French fries, Mexican, beer, high fat dairy, liquor
3. **Cluster 3:** low fat dairy, breakfast cereal, whole meal bread, fruit juice, cake, jam, ice cream
4. **Four cluster solution**
5. **Cluster 1:** low fat dairy, breakfast cereal, fruit juice, whole meal bread
6. **Cluster 2:** meat, pasta and pizza, chips, soda regular, cheese regular, confectionery, salad dressing regular, butter, French fries, Mexican, liquor
7. **Cluster 3:** cake, jam, other bread, cooked potatoes, processed meat, margarine, eggs, coffee, dessert, high fat dairy, ice cream, beer
8. **Cluster 4:** fruits, diary regular, nuts, poultry, rice, cooked vegetables, fish, soup, legumes, wine, raw vegetables, cabbage, meal replacement, olive oil
9. **Five cluster solution**
10. **Cluster 1:** meat, pasta and pizza, soda regular, chips, cheese regular, confectionery, salad dressing regular, butter, French fries, beer, ice cream, liquor
11. **Cluster 2:** low fat dairy, breakfast cereal, whole meal bread
12. **Cluster 3:** poultry, rice, fish, wine, meal replacement, Mexican, olive oil
13. **Cluster 4:** fruits, nuts, dairy regular, fruit juice, cooked vegetables, soup, legumes, raw vegetables, cabbage
14. **Cluster 5:** cake, jam, cooked potatoes, other bread, processed meat, eggs, margarine, coffee, high fat dairy
15. **Six cluster solution**
16. **Cluster 1:** whole meal bread, soup, butter, hot tea
17. **Cluster 2:** fruits, nuts, fruit juice, dairy regular, cooked vegetables, legumes, raw vegetables, cabbage
18. **Cluster 3:** meat, pasta and pizza, soda regular, chips, cheese regular, confectionery, salad dressing regular, French fries, beer, liquor
19. **Cluster 4:** jam, cake, other bread, cooked potatoes, processed meat, margarine, eggs, coffee, dessert, high fat dairy, ice cream
20. **Cluster 5:** poultry, rice, fish, wine, meal replacement, Mexican, olive oil
21. **Cluster 6:** low fat dairy, breakfast cereal
22. **Seven cluster solution**
23. **Cluster 1:** whole meal bread, cake, soup, hot tea
24. **Cluster 2:** jam, other bread, cooked vegetables, processed meat, coffee, margarine, eggs, dessert, high fat dairy
25. **Cluster 3:** breakfast cereal
26. **Cluster 4:** poultry, rice, fish, wine, meal replacement, olive oil
27. **Cluster 5:** meat, pasta and pizza, soda regular, chips, cheese regular, confectionery, salad dressing regular, French fries, beer, liquor, ice cream
28. **Cluster 6:** low fat dairy
29. **Cluster 7:** fruit, fruit juice, nuts, dairy regular, cooked vegetables, legumes, raw vegetables, cabbage
